# Acceptability, Usability, and Clinical Appropriateness of an AI-Powered Tool for Linkage to HIV Services After HIV Self-Testing in South Africa: Mixed Methods Evaluation

**DOI:** 10.2196/88570

**Published:** 2026-08-17

**Authors:** Nomsa Brightness Mahlalela, Onthatile Maboa, Caroline Govathson, Laura Rossouw, Lawrence Long, Ross Greener, Sarah Morris, Shawna Cooper, Sasha Frade, Sophie Pascoe, Candice Chetty-Makkan

**Affiliations:** 1 Department of Internal Medicine, Health Economics and Epidemiology Research Office (HE²RO), School of Clinical Medicine, Faculty of Health Sciences, University of the Witwatersrand, Sunnyside Office Park Building C, Johannesburg, 2193, South Africa, 27 769822046; 2 Department of Global Health, Boston University School of Public Health, Boston, MA, United States; 3 Product & Strategy, Audere, Seattle, WA, United States; 4 Research, Monitoring and Evaluation, Audere Africa, Johannesburg, Gauteng, South Africa

**Keywords:** acceptability, usability, digital health, artificial intelligence, HIV self-testing, South Africa

## Abstract

**Background:**

Timely linkage to HIV prevention and treatment services following HIV self-testing (HIVST) remains a challenge in many countries. While HIVST offers privacy and convenience for individuals to know their HIV status, many do not engage in follow-up care due to behavioral barriers like uncertainty about next steps, stigma, and privacy concerns.

**Objective:**

This study evaluated the acceptability, usability, and clinical appropriateness of Your Path, an AI-powered tool designed to facilitate access to HIV services following HIVST.

**Methods:**

This study was conducted with community members and health care providers, recruited from Indlela’s Behavioral Hub in South Africa. Community members were randomly assigned a mock HIV result and completed a pretest survey assessing intentions to seek HIV services, followed by a simulated HIVST guided by Your Path*,* delivered via WhatsApp (Meta Platforms). The tool provides guidance for HIVST support, interpretation of results, emotional support, and delivers tailored information to access HIV services through an interactive chat interface. Community members then completed a posttest survey assessing intentions to seek HIV services and usability of the tool using the System Usability Scale (SUS) questions. A subsample completed in-depth interviews exploring their experience with Your Path. Health care providers reviewed interaction transcripts, assessing clinical appropriateness, completeness, and relevance of the summaries; qualitative data underwent thematic analysis using the Theoretical Framework of Acceptability.

**Results:**

A sample of 100 community members aged between 18 and 61 years was enrolled: 59 (59%) were female, and 51 (51%) were assigned a negative mock HIV result. Overall, the intervention had its strongest effect on participants’ confidence in accessing HIV services, particularly among those with HIV-negative results (Wilcoxon *P*=<.001; Stuart-Maxwell *P*=.004). Intentions and perceived importance were high at baseline and remained largely unchanged over time in both groups. The tool demonstrated high usability, with a mean SUS score of 81.6 (SD 17.5) out of 100. Most found Your Path easy to use (84/100, 84%), 76 of 100 (76%) did not need assistance, and 95 of 100 (95%) expressed willingness to use it again. However, 15 of 100 (15%) noted it did not always provide consistent responses to questions. From qualitative interviews, the majority described Your Path as helpful, user-friendly, acceptable, and private. Participants appreciated its private and nonjudgmental tone and confidentiality, though some expressed information protection concerns. Twenty-five health care providers aged between 24 and 64 years participated in qualitative interviews. The majority found the summaries clinically appropriate, informative, and beneficial for HIV care linkage, but noted limited emotional responsiveness and language barriers.

**Conclusions:**

Your Path was acceptable, usable, and clinically appropriate for supporting access to HIV services after HIVST. Larger in-field studies are needed to understand the implementation, cost, and sustainability of digital interventions in public health.

## Introduction

The global HIV/AIDS epidemic continues to be a major public health challenge, with an estimated 44.1 million deaths since the start of the epidemic [1]. Transmission persists globally, affecting all countries, including South Africa. Undiagnosed HIV significantly contributes to the ongoing transmission of the virus, and early detection remains key to reaching epidemic control. HIV testing is a critical step in diagnosis and linking infected individuals to effective HIV treatment and care, and those at increased risk of acquiring HIV to prevention services. Early treatment initiation among those infected with HIV has been shown to reduce rates of HIV transmission, improve health outcomes, and reduce the cost of care [2-4]. To increase access and uptake of HIV testing, South Africa offers extensive HIV testing programs, with HIV self-testing (HIVST) included as a supplementary strategy in the National HIV Testing Services Policy in 2016, especially among key and under-tested populations [5]. HIVST is a process whereby individuals collect their own specimens, conduct an HIV test, and interpret their results in a private space.

HIVST is available in many countries, including South Africa, offering individuals a discreet and convenient method to learn their HIV status. However, many individuals who self-test do not proceed with confirmatory testing if the result is positive as required by the World Health Organization [6,7], or access preventive services such as preexposure prophylaxis (PrEP) if the result is negative. Limited linkage to care following HIVST is often due to uncertainty about next steps, stigma and discrimination, fear of negative attitudes from health care providers, concerns about privacy, or structural barriers such as cost and time constraints [8,9]. Effective linkage to confirmatory testing, HIV treatment, care, and prevention, including PrEP, is therefore crucial following HIVST, particularly those conducted in home settings where direct support and referral from health care providers are not immediately available.

AI is evolving to support health decision-making and presents potential to address behavioral barriers to accessing appropriate HIV care and prevention services. In health care settings, the transformative role of generative AI models is increasingly being recognized for their ability to enhance health education, deliver perceived empathetic support, and manage data [10]. For instance, large language models (LLMs), including ChatGPT and Claude, can engage with users by answering health-related questions, offering tailored guidance, and facilitating linkage to care using destigmatizing language [11,12]. A narrative review of chatbot applications in HIV prevention and care found that conversational tools can support sensitive discussions about HIV, promote prevention and treatment engagement, and are influenced by users’ perceptions of trustworthiness, accuracy, and privacy [11]. Similarly, a systematic review of HIVST interventions with digital support strategies reported improved uptake, strong participant acceptability, and promising linkage-to-care outcomes across key and general populations, particularly among first-time testers and hard-to-reach groups [12].

More broadly, AI applications are being leveraged to strengthen public health responses. For example, predictive analytics is being used to forecast disease outbreaks, identify high-risk populations, and inform targeted interventions and resource allocation [13,14]. In Kenya, AI tools have been deployed across 1800 health care facilities to enhance HIV case finding and tailor prevention strategies to individuals at the highest risk [15]. AI tools that integrate computer vision and conversational agents are also being scaled up across sub-Saharan Africa to support HIVST by enabling real-time interpretation of test results and facilitating reflexive linkage to appropriate services [16,17]. These innovations demonstrate how AI can help close gaps in the HIV care continuum by offering more responsive, scalable, and client-centered approaches. However, despite growing evidence on how digital tools can support HIVST and linkage to prevention or treatment, there remains limited empirical evidence on how generative AI–enabled conversational tools can support post-HIVST counseling interactions and influence care-seeking intentions, especially in low- and middle-income countries (LMICs) where the burden of HIV is highest. Given South Africa’s high HIV prevalence, widespread mobile phone access, and evolving digital health infrastructure, there is a critical opportunity to evaluate the potential of AI-enabled tools to improve linkage to care and prevention services following HIVST in ways that are private, personalized, and scalable.

Building on Your Choice, an earlier AI-powered tool for HIV risk assessment and personalized prevention guidance, Your Path was developed to engage users in post-HIVST conversations via an AI-powered interactive WhatsApp-based chat. Your Path provides tailored guidance and support on next steps for accessing HIV services following HIVST. In this evaluation, the AI component was used to generate conversational responses to user messages during the posttest chat and to support the creation of conversational summaries for health care provider review. The objective of this study was to evaluate the acceptability, usability, and clinical appropriateness of Your Path in facilitating engagement with HIV services following a simulated HIVST experience in South Africa.

## Methods

### Study Design and Setting

This exploratory evaluation used quantitative and qualitative methods and was conducted with community members and health care providers recruited from Indlela’s Behavioral Hub (B-Hub) in Gauteng province, South Africa. The B-Hub is a research initiative focused on testing behavioral interventions for HIV prevention and care [18]. The hub is located at the Health Economics & Epidemiology Research office (HE^2^RO) in Johannesburg, a large urban metropolitan area. Participants were recruited from urban and semiurban communities across the province. Despite being the smallest province in South Africa, Gauteng is the most densely populated province with an estimated population of 16.1 million people. The province is highly urbanized and serves as the country’s economic center, attracting people from different social and economic backgrounds. While Gauteng includes some peri-urban and informal settlement areas, it is predominantly urban compared to other provinces in South Africa.

### Recruitment and Sampling

Participants were recruited from the B-Hub database, which served as the sampling frame. The B-Hub maintains a consented research participants panel of community members and health care providers from Gauteng province who agreed to be contacted for future research studies. We used purposive sampling to ensure inclusion of eligible community members who met the study eligibility criteria and health care providers with experience in HIV service delivery. Community members and health care providers were contacted telephonically by trained research staff to assess interest and confirm eligibility. Eligible community members were adults aged ≥18 years who were willing to provide written informed consent in English, self-reported an HIV-negative or unknown HIV status, and were able to travel to the study site. Individuals who self-reported as HIV-positive were excluded. Eligible health care providers included professional nurses, HIV counseling and testing (HCT) counselors, and health coaches, as these cadres are directly involved in HIV testing, prevention counseling, linkage to care, and ongoing patient support within routine HIV service delivery. Health care providers were eligible if they were able to provide informed consent in English and had at least one year of experience providing HIV services. Recruitment and data collection took place between November 2024 and January 2025. Data collection with community members was conducted at the Indlela@HE^2^RO research office, while interviews with health care providers were conducted at their respective clinics. Only the researcher and the research assistants were present during data collection. As this was a rapid prototype pilot evaluation, sample sizes were determined pragmatically to assess usability, acceptability, and perceived appropriateness of the tool among intended users rather than to test effectiveness outcomes.

### Study Procedures

Eligible community members were randomly assigned to a mock HIV-negative or positive test result scenario prior to the simulated HIV self-test and interaction with the Your Path tool. Participants were asked to envision themselves in their assigned scenario when completing study activities and interacting with the tool. This approach was chosen because the study’s primary aim was not to replicate real-world HIVST procedures, but to evaluate the usability and acceptability of the AI tool. Providing the mock result beforehand allowed participants to engage fully with the Your Path tool, which offers step-by-step guidance on performing the test and tailored recommendations through a chat with an AI companion for next steps in accessing HIV services based on their assigned result.

Community members then completed a pretest baseline survey (Table S1 in Multimedia Appendix 1). They were then provided with study phones and instructed to open the Your Path WhatsApp thread by typing “hi,” which triggered an automated welcome message and menu options. From the main menu, participants selected the “Self-test support” option and completed a simulated HIVST using the Mylan HIVST kit (Viatris) option, guided step-by-step by Your Path. Your Path AI-Companion is a rule-guided conversational chatbot designed to simulate post–HIVST counseling and linkage support. The tool provides structured information and conversational guidance across several topics, including interpreting HIV self-test results, emotional support following testing, HIV treatment initiation and linkage to care (for positive results), HIV prevention options including PrEP (for negative results), and planning next steps for accessing services. Upon completion of the simulated HIVST, participants were prompted to discuss their assigned mock test results with the Your Path AI-companion. The posttest chat provided an opportunity for the participant to ask free-text questions, with the tool responding in a conversational style to discuss the participant’s mock result and next steps. During the chat, the AI-companion posed context-specific questions including (1) support interpreting HIV self-test results; (2) recommended next steps and linkage guidance (eg, confirmatory testing and treatment linkage after a positive result; prevention and repeat testing guidance after a negative result); (3) emotional support and normalization of common concerns; and (4) responses to common questions about HIVST and services. We structured the sessions to provide about 10‐15 minutes for participants to engage in the posttest chat. Additionally, chat transcripts were logged for qualitative analysis, and participants consistently completed the posttest chat component as part of the simulated workflow. Chats occurred in a private space to ensure participant comfort and confidentiality. Participants were asked not to enter any personal identifying information. All interactions were conducted using study access accounts, and chatbot interaction logs were securely recorded for analysis, including conversation length and number of message exchanges.

After engaging with Your Path, community members completed the posttest survey (Table S2 in Multimedia Appendix 1), which also included validated System Usability Scale (SUS) questions [19], to measure the usability of Your Path. A subset of community members was then purposively selected for semistructured interviews to explore the acceptability of Your Path features, as well as barriers and motivators to engagement. To allow for variation, community members were purposively selected based on age, gender, and mock test results (negative or positive). The interviews were audio recorded and included prompts (Table S3 in Multimedia Appendix 1) regarding the community member’s experience using Your Path and suggestions for improvement. Each interview lasted about 20 minutes on average and was conducted in a private room where only the participant and the interviewer were present. The interviews were conducted by 2 female study team members (NM and OM) with master’s degrees who are experienced researchers. The sample size was adequate to see a recurrence in themes, and data saturation was assessed during the data collection process through frequent revision of the probes.

Health care providers engaged in 2 steps. First, they reviewed 2 transcripts of interactions between community members and Your Path together with the AI-generated summaries corresponding to those same transcripts. Secondly, they participated in semistructured interviews, each lasting approximately 30 minutes. During interviews, health care providers examined whether the tool provided clear and accurate guidance on next steps after an HIV self-test, offered appropriate emotional support, and facilitated posttest counseling conversations. Health care providers also evaluated the completeness and relevance of the AI-generated summaries, including whether the summaries captured key user concerns and counseling needs reflected in the transcripts.

### Tool Development

Development of the Your Path conversational content and decision pathways was informed and grounded with a knowledge base of World Health Organization guidelines, South African national guidelines, and other relevant local-specific HIV testing and posttest counseling guidelines to structure the conversational goals and recommended next steps. The AI-companion is powered by LLMs, using prompt engineering techniques and referencing the knowledge base to improve the consistency and appropriateness of counseling-style responses, support capture of key data elements (eg, HIV status) from each conversation to maintain continuity and ground context across subsequent chat interactions. Prior to the study evaluation, iterative prestudy testing was conducted that included: (1) scenario testing using simulated user conversations representing expected HIVST outcomes and common user concerns; (2) pilot testing of the WhatsApp-based interaction flow to confirm usability and technical performance; and (3) review of simulated transcripts to assess alignment with guideline-recommended counseling, clarity of next steps, and appropriateness of tone and supportive language. Findings from these activities informed the refinement of the conversation flow and response behavior before deployment.

### Data Analysis

Statistical analyses were conducted using Stata 18 (StataCorp LLC) [20]. Descriptive statistics, including percentages, means, medians, and SDs, were used to summarize sociodemographic variables and survey responses. To assess changes in participants’ intentions to engage in HIV-related services, we compared paired pre- and postintervention responses across a series of Likert-scale outcomes. These outcomes captured intentions, confidence, and perceived importance of engaging in HIV prevention or treatment services, depending on mock HIV test results. Results were not compared across HIV test result scenarios (HIV-negative vs. HIV-positive), as the underlying questions were not directly comparable: HIV-negative scenarios referred to engagement in prevention services, whereas HIV-positive scenarios referred to engagement in treatment services. While the results of the intention to engage in HIV services before and after using the intervention are reported, it should be noted that the study was not powered to measure efficacy, and results should therefore be interpreted with caution.

Given the ordinal nature of the responses, we used two complementary nonparametric approaches to test for statistical significance pre- and post-interacting with the Your Path intervention. Firstly, we used the Wilcoxon signed-rank test to evaluate within-individual changes over time, testing whether there was a systematic directional shift in responses. Secondly, we applied the Stuart-Maxwell test to assess whether the overall distribution of responses across categories differed between pre- and postintervention. This provides a complementary assessment of whether responses were redistributed across categories, irrespective of direction. Statistical significance was assessed at the 5% level (*P*<.05). Community members’ perception of the usability of Your Path was assessed using the SUS, a validated 10-item questionnaire in which each item is rated on a 5-point Likert scale [21]. A mean SUS score of 68 or higher is considered above average, while a score of 80 or above indicates excellent usability. However, interpretation of SUS scores can vary based on the system’s complexity, target audience, and context of use.

Qualitative data were analyzed using NVivo (version 14, Lumivero). Audio recordings were transcribed using a professional transcription service. Thematic analysis using a deductive approach was used to derive and analyze themes from the data. Codebooks were developed and refined through an iterative approach by three researchers (NM, CG, and OM). During discussions, interrater reliability across themes was assessed where similar themes were retained while others were dropped when no consensus was reached. Qualitative findings are reported in accordance with the Consolidated Criteria for Reporting Qualitative Research (COREQ) guidelines (Table S4 in Multimedia Appendix 1). The study results were aligned with the Theoretical Framework of Acceptability (TFA) [22]. We did not stratify themes by HIV result because the study was designed to explore overall acceptability, usability, and clinical appropriateness, rather than to compare experiences by HIV status. Additionally, the small number of participants interviewed limits meaningful stratified analysis.

### Ethical Considerations

The Wits Human Research Ethics Committee (medical) approved the study (reference number 211122). Written informed consent was obtained from all participants prior to their participation in the study. All identifying information has been removed or anonymized to protect participants' privacy and maintain confidentiality.

## Results

### User Statistics

Participants included community members and health care providers. A total of 154 community members were invited to participate, of whom 100 were enrolled. Screening failures (n=54) were primarily due to inability to travel to the site, work schedule conflicts, and a lack of interest in participating. Similarly, 37 health care providers were invited, with 25 enrolled; screen failures (n=12) were largely due to scheduling conflicts or not meeting inclusion criteria. Table 1 presents the sociodemographic characteristics of the enrolled community members and health care providers. The median age was 28.8 (IQR 18‐61) years for community members and 37.5 (IQR 24‐61 years for health care providers. 59 out of 100 (59%) community members were female, 92 out of 100 (92%) self-reported HIV-negative, and 55 out of 100 (55%) were unemployed. Among health care providers, 21 out of 25 (83%) were female, 15 out of 25 (60%) were experienced HCT counselors, and 18 out of 25 (81%) had been employed for more than three years.

**Table 1.** Community members and health care providers sample characteristics (n=125).

Variables	Community members n=100 (%)	Health care providers n=25 (%)
Age (years), median (IQR)	28.8 (IQR 18‐61)	37.5 (IQR 24‐61)
Sex		
Male	41 (41)	4 (16)
Female	59 (59)	21 (84)
Self-reported HIV status		
HIV negative	92 (92)	—^a^
Did not disclose	8 (8)	—
Employment status		
No	55 (55%)	—
Yes	45 (45%)	—
Profession		
Professional nurse	—	9 (36)
HCT^b^ counselor	—	15 (60)
Health coach	—	1 (4.0)
Years of employment in current profession		
Less than 6 months	—	1 (4.0)
1‐3 years	—	6 (24.0)
4 years and above	—	18 (72.0)

a Not applicable.

b HCT: HIV counseling and testing.

### Evaluation Outcomes

Chatbot interaction logs showed active engagement with the Your Path AI-Companion. On average, participants interacted with the AI-Companion for 15 minutes (range: 10‐15). Most participants asked at least 5 free-text questions during the interaction, in addition to responding to structured prompts. These interactions reflected conversational engagement similar to a posttest counseling session. For participants with mock HIV negative test results, there was limited evidence of change in intentions to engage in HIV prevention services following the intervention (Table 2). Perceived importance of engaging in prevention services remained high at both timepoints, with no statistically significant change (Wilcoxon *P*=.79; Stuart-Maxwell *P*=.38). Similarly, intentions to seek services if testing positive and confidence in accessing resources showed no significant shifts, although confidence in accessing prevention services increased significantly (Wilcoxon *P*=.0002; Stuart-Maxwell *P*=.0039), suggesting both a directional improvement and a redistribution toward higher confidence categories. A marginal improvement was observed in confidence to find resources (Wilcoxon *P*=.012), although this was not accompanied by a significant change in the overall distribution (Stuart-Maxwell *P*=.16). No significant changes were observed in the reported likelihood of engaging in prevention or treatment services.

**Table 2.** Intention to seek HIV services for community members assigned negative mock test result (n=51).

	Pretest, n (%)	Posttest, n (%)	Wilcoxon signed-rank test, *P* value	Stuart-Maxwell-type test, *P* value
Engaging in HIV prevention services is beneficial for my health			.79	.38
Strongly disagree	6 (12.2)	6 (12.8)		
Somewhat disagree	3 (6.1)	—^a^		
Neither agree nor disagree	2 (4.1)	3 (6.4)		
Somewhat agree	2 (4.1)	3 (6.4)		
Strongly agree	36 (73.5)	35 (74.5)		
Seeking HIV treatment services is important if I test positive			.06	.19
Strongly disagree	6 (12.2)	4 (8.5)		
Somewhat disagree	2 (4.1)	2 (4.3)		
Neither agree nor disagree	3 (6.1)	1 (2.1)		
Somewhat agree	3 (6.1)	—		
Strongly agree	35 (71.4)	40 (85.1)		
I am confident that I can access HIV prevention services if I need to			*P*<.001	.004
Not confident at all	—	—		
Not confident	1 (2.0)	—		
Unsure	3 (6.1)	1 (2.1)		
Confident	28 (57.1)	19 (40.4)		
Very confident	17 (34.7)	27 (57.4)		
I am confident that I can find the resources (eg, money and time) to seek HIV treatment services if I test positive			.01	.16
Not confident at all	2 (4.1)	1 (2.1)		
Not confident	3 (6.1)	2 (4.3)		
Unsure	6 (12.2)	5 (10.6)		
Confident	23 (46.9)	17 (36.2)		
Very confident	15 (30.6)	22 (46.8)		
How likely are you to engage in HIV prevention services?			.54	.07
Extremely likely	15 (30.6)	21 (44.7)		
Very likely	25 (51.0)	16 (34.0)		
Somewhat likely	4 (8.2)	4 (8.5)		
Very unlikely	5 (10.2)	3 (6.4)		
Extremely unlikely	—	3 (6.4)		
How likely are you to seek future HIV treatment services if you test HIV positive?			.11	.51
Extremely likely	25 (51.0)	30 (63.8)		
Very likely	15 (30.6)	12 (25.5)		
Somewhat likely	2 (4.1)	1 (2.1)		
Very unlikely	4 (8.2)	3 (6.4)		
Extremely unlikely	3 (6.1)	1 (2.1)		

a Not applicable.

For individuals with a positive HIV mock test result, the perceived importance of seeking treatment services remained consistently high, with no significant change between pre- and postintervention responses (Wilcoxon *P*=.68; Stuart-Maxwell *P*=.67; Table 3). Confidence in the ability to find resources to seek treatment services showed a statistically significant directional increase (Wilcoxon *P*=.04), although the overall distributional change was not statistically significant (Stuart-Maxwell *P*=.10), indicating a modest upward shift within categories rather than a broad redistribution. No significant changes were observed in the stated likelihood of engaging in treatment services. Overall, the intervention appears to have had its strongest effect on participants’ confidence in accessing HIV-related services, particularly in the HIV-negative (prevention) context, while intentions and perceived importance were already high at baseline and remained stable over time.

Following interaction with Your Path, its usability was rated highly, achieving a mean SUS score of 81.6 (SD 17.5; Table 4). Most community members (84/100, 83.7%) found the tool easy to use, 94.9% (95/100) would use it again, and 90.8% (91/100) felt confident using it. However, 33,6% (34/100) preferred assistance before using the tool, and 15.3% (15/100) noted that it did not always provide consistent responses to questions where they felt that responses to some queries varied or were unclear.

The deductive themes developed in accordance with the TFA and derived from the in-depth interviews are presented below and summarized in Table 5.

**Table 3.** Intention to seek HIV services for community members assigned positive mock test result (n=49).

	Pretest, n (%)	Posttest, n (%)	Wilcoxon signed-rank test, *P* value	Stuart-Maxwell-type test, *P* value
Seeking HIV treatment services is important			.68	.67
Strongly disagree	6 (11.5)	6 (11.8)		
Somewhat disagree	1 (1.9)	—^a^		
Neither agree nor disagree	—	1 (2.0)		
Somewhat agree	1 (1.9)	—		
Strongly agree	44 (84.6)	44 (86.3)		
I am confident that I can find the resources (eg, money and time) to seek HIV treatment services			.04	.10
Not confident at all	5 (9.6)	4 (7.8)		
Not confident	4 (7.7)	4 (7.8)		
Unsure	5 (9.6)	5 (9.8)		
Confident	23 (44.2)	13 (25.5)		
Very confident	15 (28.8)	25 (49.0)		
How likely are you to seek HIV treatment services?			.60	.78
Extremely likely	26 (50.0)	28 (54.9)		
Very likely	16 (30.8)	15 (29.4)		
Somewhat likely	4 (7.7)	3 (5.9)		
Very unlikely	3 (5.8)	1 (2.0)		
Extremely unlikely	3 (5.8)	4 (7.8)		

a Not applicable.

**Table 4.** System Usability Scale (SUS; N=100).

SUS item	SUS score (mean 81.6, SD 17.5, median 87.5, IQR 75-95)				
	Strongly disagree, n (%)	Somewhat disagree, n (%)	Neither agree nor disagree, n (%)	Somewhat agree, n (%)	Strongly agree, n (%)
I think that I would like to use the Your Path chat when I want to self-test for HIV.	4 (4.1)	0 (0.0)	1 (1.0)	11 (11.2)	84 (83.7)
I found the Your Path chat difficult to understand.	67 (68.4)	11 (11.2)	6 (6.1)	4 (4.1)	10 (10.2)
I thought the Your Path chat was easy to use.	7 (7.1)	2 (2.0)	7 (7.1)	11 (11.2)	71 (72.5)
I think that I would need help to be able to use the Your Path chat.	49 (50.0)	12 (12.2)	4 (4.1)	8 (8.2)	25 (25.5)
I think the different features in the Your Path chat worked well and felt connected from start to finish. work really well together.	3 (3.1)	1 (1.0)	3 (3.1)	11 (11.2)	80 (81.6)
I think the Your Path chat is confusing because it doesn’t always give the same answers or respond the same way to a question.	62 (63.3)	16 (16.3)	5 (5.1)	6 (6.1)	9 (9.2)
I imagine that most people would learn to use the Your Path chat very quickly.	5 (5.1)	3 (3.1)	2 (2.0)	14 (14.3)	74 (75.5)
I found the Your Path chat very difficult to use.	80 (81.6)	4 (4.1)	0 (0.0)	4 (4.1)	10 (10.2)
I felt very confident using the Your Path chat.	2 (2.1)	3 (3.1)	2 (2.1)	6 (6.2)	84 (84.6)
I needed to learn a lot of things before I could start using the Your Path chat.	36 (36.7)	15 (15.3)	8 (8.2)	16 (16.3)	23 (23.5)

**Table 5.** Deductive themes aligned with the Theoretical Framework of Acceptability. (Adapted from Sekhon et al, 2017).

TFA^a^ construct	Summary of findings
Affective attitude	Community members generally described interactions with Your Path as warm, supportive, and nonjudgmental, which made discussing HIV testing and care easier. In contrast, health care providers reviewing transcripts felt the chatbot interaction lacked emotional depth and empathy compared to in-person counseling.
Burden	Both community members and health care providers expressed concerns about potential burdens, including privacy, language accessibility (particularly vernacular options), and barriers related to mobile data and internet access.
Ethicality	Community members perceived Your Path as less judgmental than clinic-based counseling, allowing them to ask sensitive questions more freely. This contributed to feelings of comfort and psychological safety when discussing HIV-related concerns.
Intervention coherence	Community members consistently described the tool as easy to use, simple to understand, and responsive, indicating that they understood how the intervention worked and how to interact with it.
Perceived effectiveness	Community members and health care providers generally perceived the tool as effective, noting that it provided useful step-by-step guidance on HIV self-testing, prevention (including PrEP^b^), and treatment linkage. Health care providers noted its potential value for individuals reluctant to visit clinics due to stigma.

a TFA: theoretical framework of acceptability.

b PrEP: preexposure prophylaxis.

### Feelings Expressed When Engaging With Your Path and Reviewing Transcripts (Affective Attitude)

Community members described the interactions with Your Path as warm, welcoming, and offering personalized support that was comforting and reassuring.

*It felt like I was talking to somebody. So, for me I felt like the app, you talking to another human being … like it was so warm, it was full of emotions.*

[Female, 35 years, negative mock results]

*It made me feel better. You know that as a man it is not easy to talk to people. Your Path made things simpler.*

[Male, 30 years, positive mock results]

However, when health care providers were reviewing the transcripts and summaries, 5 out of 25 (20%) felt that Your Path did not provide adequate precounseling *and the interaction seemed robotic, lacking the warmth and responsiveness of human interaction.*

*When you do counselling with a patient, you become more empathetic with the patient, so when you're using the chatbot….you cannot reach that level with the patient. So, when it’s face to face and you're there with the patient, you can see their emotions through their eyes and their body language. When using the chatbot you can’t. You just answer whatever that they are texting.*

[Female, 40 years, health care provider]

*I feel because it’s, it’s AI man, it’s impersonal. It doesn't give an opportunity to … the client to express their feelings…*

[Male, 37 years, health care provider]

### Concerns Expressed About Your Path (Burden)

Six out of 25 (24%) community members and 10 out of 25 (40%) health care providers raised concerns about privacy, language limitations, and overall accessibility of Your Path.

*…I don’t believe that there will be privacy or something…I had the concern, that one can see it …*

[Male, 38 years, negative mock results]

*I think they should add more pictures and be able to use the vernacular, …because some people do not know English that well.*

[Female, 40 years, health care provider]

*Only a few things people would complain about is data… .and maybe access to Wi Fi.*

[Male, 37 years, health care provider]

### Comparison of a Digital Platform Versus in-Person Interactions for Counseling (Ethicality)

Community members perceived interactions with Your Path as friendly and nonjudgmental when compared to engaging with health care providers during counseling sessions.

*…I won't get any judgmental features, I can say whatever … and it won’t give me any judgment at the end of the day… People are judgemental…It is much better with Your Path.*

[Male, 25 years, negative mock results]

*When being answered there, like I was not being judged … if they had said I should go to the clinic, those questions that I was asking through the Your Path, I don’t see, I would have asked them, a sister like while looking at her cause hey! They judge you.*

[Female, 43 years, positive mock results]

### Community Member’s Ability to Understand and to Use Your Path (Intervention Coherence)

Community members described Your Path as user-friendly, simple, and convenient to use, enabling straightforward access to information and support.

*So, much simple, it is simple this thing…it responds and quick.*

[Female, 46 years, positive mock results]

*…It’s much easier to use … Al, so it’s so much easier for you to understand what it refers you to and what it says to you, I think, with your direct question.*

[Male, 22 years, positive mock results]

### Perceived Benefits of Your Path for HIV Services (Perceived Effectiveness)

Community members and health care providers felt that Your Path provided useful and relevant step-by-step guidance for HIV self-testing and appropriate information on HIV prevention and treatment, supporting linkage to appropriate HIV services. Health care providers also viewed it as a helpful tool for individuals hesitant to visit clinics.

*I gained a lot of knowledge…I didn’t know about PrEP. That PrEP are pills that prevent you from getting HIV, …I didn’t know that if you take your medication well, while positive, you are able to not infect other people…*

[Female, 25 years, positive mock results]

*What I liked is that the AI… was talking to the participant that he must make sure he goes to the nearest clinic to confirm the result that he or she got …*

[Female, 40 years, health care provider]

*There are people who will need help, but they're afraid because of stigma. So, I think this AI is a good thing, because a person can go to her room or his room alone and do this, and then they know their status … if they listen to the AI to go and look for assistance at the nearest clinic.*

[Female, 40 years, health care provider]

## Discussion

### Principal Findings

In this controlled exploratory study, Your Path*,* an AI-powered tool designed to facilitate access to HIV prevention and treatment services following HIVST, showed limited change in intentions to engage with HIV services, with intentions remaining high pre and post intervention. Most community members found the tool easy to use and appreciated its private and nonjudgmental tone, which helped reduce perceived stigma. Health care providers considered the tool to be clinically appropriate, informative, and relevant for supporting access to HIV care, although some limitations were noted regarding emotional responsiveness and language accessibility. To our knowledge, this study is among the first conducted in a resource-limited setting to evaluate a digital AI-powered technology specifically focused on facilitating post-HIVST linkage to care, highlighting the potential of AI-powered interventions to address gaps in HIV service delivery. These findings underscore the promise of user-centered digital tools to support timely connection to care, particularly in contexts where access to health care resources is constrained.

### Comparison With Prior Work

Most studies that evaluated the use of digital tools for public health services have been conducted in high-income countries with well-established digital infrastructure [23-26]. Although recent research in Africa has begun to explore the role of mobile health (mHealth) interventions, such as SMS reminders and chat-based platforms, in improving HIV testing uptake, medication adherence, and clinic appointment reminders [27-33]. Despite progress in research evaluating the integration of digital tools into public health, few studies have specifically examined how AI-powered conversational agents can support and strengthen linkage to HIV prevention and treatment services in South Africa. Our study contributes to closing this gap by evaluating the usability, acceptability, and clinical appropriateness of the AI-powered Your Path tool, designed to facilitate linkage to HIV prevention and treatment services following HIVST in South Africa. Most studies exploring the use of digital tools in public health focused primarily on the perspective of clients [25,26,28,29], while our study included both the perspectives of clients and health care providers to capture a comprehensive understanding.

Within HIVST programs in sub-Saharan Africa, linkage to care following an HIVST is poor, and this is attributed to behavioral barriers such as stigma and lack of guidance on the next steps. For example, the Tanzania HIV self-testing education and promotion project targeted men to improve HIV testing uptake [34], and a program in central Uganda offered HIV oral self-testing to male partners of women attending antenatal care [35]; both studies reported challenges in ensuring linkage to care. AI tools present an opportunity to address barriers, particularly through the application of digital conversational agents. Studies have shown that AI-based conversational agents are acceptable within an HIV context and can engage users on the next steps post HIVST in a manner that is private, confidential, and nonjudgmental [28]. For instance, studies have shown that these features make conversational agents especially valuable for high-risk populations, offering stigma-free entry points into HIV services [36,37]. Our study findings align with this, where community members expressed a preference for engaging with the Your Path AI companion over a real counselor due to fear of stigma, judgment,w and perceived lack of confidentiality with human interaction. These findings are also consistent with results from another study [38], where users often preferred digital health care agents over human providers when discussing sensitive issues.

Our findings also highlight important divergent views between community members’ and health care providers’ perspectives of the AI companion. While community members valued the AI companion’s nonjudgmental tone, privacy, and accessibility, a few health care providers raised concerns about the quality of interactions. They described these interactions as impersonal and felt that the AI companion was less able to provide emotional support compared to human counseling. These differing perspectives may reflect differences in expectations, with providers emphasizing relational and empathetic aspects of counseling as core components of care, while community members value nonhuman interactions, as they allow for more open discussion of sensitive issues without fear of judgment. Despite the health care providers’ concerns about the quality of interactions, other studies have shown that patients preferred to use AI over talking to a human provider, as they deemed it more empathetic as compared to a human health care provider [39,40]. To address the health care providers’ concerns, tools such as Your Path could be provided as options alongside in-person human interaction so community members can choose whether they want to interact with chatbots or health care providers. For future implementation of AI tools such as Your Path in health care, it’s important to position AI-powered tools as complementary tools to provider-delivered counseling.

Our findings indicate that Your Path is acceptable, which aligns with studies conducted in Malaysia [35] and South Africa [28,41,42] as well as a broader scoping review [43], which show that digital tools integrated with AI are particularly well-received in the context of HIV care. Acceptability of digital interventions is influenced by multiple factors, including usability, confidentiality, and emotional support [44]. In our study, there was a high willingness to use Your Path, reflecting openness to incorporating digital technologies into HIV self-testing and posttest support. Qualitative interviews highlighted emotional safety and confidentiality as key contributors to user satisfaction. Many people living with HIV still face significant psychological challenges as they are often discriminated against, socially isolated, and stigmatized for their condition [45-47]. Our findings, along with those of other studies [39,45], suggest that a non-judgmental, anonymous conversational agent like Your Path can provide a reassuring and safe space for users to discuss sensitive topics, such as HIV self-testing results, without fear of being criticized*.* This may encourage users to seek information freely and might enhance their willingness to use Your Path*.*

Our findings also highlight the strong usability of Your Path, with a mean usability score of 81.6/68 (SD 17.5), exceeding the preset threshold for success. Participants reported high ease of use and described the tool as accessible and simple to navigate, consistent with other studies showing the value of digital interventions when designed for familiarity and convenience [48,49]. The predominance of young participants in our sample may have contributed to these positive perceptions, given the widespread use of WhatsApp and uptake of mobile technology among youth, consistent with previous studies [28,50,51]. Together, these findings suggest that Your Path is well-positioned to engage users by combining high acceptability with strong usability, both of which are critical for sustaining adoption of digital health tools in HIV care.

### Strengths and Limitations

Several concerns and limitations, including data privacy, security of personal information, and data access, were highlighted despite positive perceptions of Your Path, a finding consistent with other studies on digital health interventions in sub-Saharan Africa [52-54]. To align with the South African Protection of Personal Information Act (2013) [55] and strengthen user trust, future versions of Your Path could implement explicit user consent processes, minimize the collection of personal identifiers, and provide users with the option to delete their data. The reliance of the current version of Your Path on mobile data and the exclusive use of English are limitations that could hinder accessibility, particularly for users in low-connectivity areas or those not proficient in English. Addressing these challenges may require offering data-free access, a strategy already used in South African mHealth programs such as Mom Connect, B-Wise, and the Moya app, which expanded reach by eliminating data costs for end-users [56-58]. To address language limitations, future versions of Your Path could include other local and regional languages to increase reach and access.

This study has contributed important information to the acceptability, usability, and clinical appropriateness of AI-powered tools for HIV care in South Africa, but it has limitations. Recruitment was limited to a single urban site, with predominantly younger participants who are more likely to have access to and be comfortable with digital technologies. As a result, our findings may not be fully generalizable to rural communities and older populations where access to and familiarity with such technologies may differ. The limitations in our study underscore the importance of future field-based evaluation with a more diverse population. Such studies will be essential to assess the acceptability, usability, and clinical appropriateness of Your Path in a real-life context where logistical, behavioral, and system-level factors play a larger role. We conducted our study in a controlled simulated environment. However, the simulated environment was necessary to allow for safe, ethical testing of feasibility before moving to real-world implementation. This evaluation did not develop or validate a clinical prediction model; therefore, TRIPOD or TRIPOD-AI performance reporting (eg, discrimination, calibration, and predictor-weight specification) was not applicable to the aims of this acceptability, usability, and clinical appropriateness assessment. A further limitation is that the qualitative results reflect the overall usability experiences of the total sample and are not separated by HIV assigned status. This limits the ability to identify potential differences in experiences or needs between participants who received a negative or a positive HIV self-test result. Future research could be conducted to understand the effectiveness of this app for achieving health outcomes for individuals with reactive and nonreactive self-test. Strengths of this study include its mixed methods design, which allowed for a comprehensive, user-centered evaluation of acceptability, usability, and clinical appropriateness. By incorporating direct input from both community members and health care providers, the study ensured that Your Path was evaluated in alignment with the real-world needs and preferences of its end users.

### Conclusions

In conclusion, this study found Your Path to be usable, acceptable, and clinically appropriate as a digital health tool to support linkage to services after an HIVST. Participants valued its ability to provide convenient access to accurate information, step-by-step guidance, and personalized support for connecting with HIV services. Insights from this study will inform future builds and adaptations of Your Path to enhance user experience, improve integration within health systems, and ensure responsiveness to emerging needs in HIV prevention and treatment. Future studies should evaluate Your Path in clinical settings to determine its real-world effectiveness, and conduct cost-effectiveness analyses to assess the value, scalability, and sustainability of AI-powered digital health tools for supporting HIV care and broader health service delivery.

## Supplementary material

10.2196/88570 Multimedia Appendix 1 Supplementary material.

## Abbreviations

B-Hub: Behavioral Hub
COREQ: Consolidated Criteria for Reporting Qualitative Research
HCT: HIV counseling and testing
HE^2^RO: Health Economics & Epidemiology Research office
HIVST: HIV self-testing
LLM: large language model
LMIC: low- and middle-income country
mHealth: mobile health
PrEP: pre-exposure prophylaxis
SUS: System Usability Score
TFA: Theoretical Framework of Acceptability
